# Lived Experience–Led Research Agenda to Address Early Death in People With a Diagnosis of a Serious Mental Illness: A Consensus Statement

**DOI:** 10.1001/jamanetworkopen.2023.15479

**Published:** 2023-05-01

**Authors:** Karen L. Fortuna, Stephanie Lebby, Pamela Geiger, Diane Johnson, Sandi MacDonald, Ilana Chefetz, Joelle C. Ferron, Lisa St George, Rebecca Rossom, Joseph Kalisa, Tomislav Mestrovic, Joanne Nicholson, Willie Pringle, Armando J. Rotondi, Lauren M. Sippel, Amie Sica, Maria E. Solesio, Maggie Wright, Yaara Zisman-Ilani, David Gambee, Julia Hill, Alison Brundrett, Corinne Cather, Taeho Greg Rhee, Gail L. Daumit, Jessica Angel, Ian Manion, Patricia E. Deegan, Jason A. Butler, Nakristia Pitts, Denise E. Brodey, Aaron M. Williams, Joseph Parks, Brie Reimann, J. Todd Wahrenberger, Oscar Morgan, Daniel W. Bradford, Nicole Bright, Elizabeth Stafford, Andrew R. Bohm, Tracy Carney, Claver Haragirimana, Alisa Gold, Marianne Storm, Robert Walker

**Affiliations:** Geisel School of Medicine, Department of Psychiatry, Dartmouth College; Concord, New Hampshire, Collaborative Design for Recovery and Health, Nashua, New Hampshire; Collaborative Design for Recovery and Health, Nashua, New Hampshire , College of Nursing and Health Sciences, The University of Vermont, Burlington, Vermont; Geisel School of Medicine, Department of Psychiatry, Dartmouth College; Concord, New Hampshire , Collaborative Design for Recovery and Health, Nashua, New Hampshire; Optum/UnitedHealthcare, Minneapolis, Minnesota; Collaborative Design for Recovery and Health, Nashua, New Hampshire , International Association of Pre-Menstrual Disorders, Boston, Massachusetts; The Hormel Institute, University of Minnesota, Austin, Minnesota; Geisel School of Medicine, Department of Psychiatry, Dartmouth College; Concord, New Hampshire; OPROMAMER, Rwanda, Africa; HealthPartners Institute, Minneapolis, Minnesota; OPROMAMER, Rwanda, Africa, University of Rwanda, Rwanda, Africa; Institute for Health Metrics and Evaluation, University of Washington, Seattle, Washington, Department of Health Metrics Sciences, University of Washington School of Medicine, Seattle, Washington, University North, University Centre Varazdin, Varazdin, Croatia; The Heller School for Social Policy and Management, Brandeis University, Waltham, Massachusetts; Centerstone, Clarksville, Tennessee; Center for Behavioral Health, University of Pittsburgh, Pittsburgh, Pennsylvania , Center for Health Equity Research and Promotion, Veterans Affairs Pittsburgh Healthcare System, Pittsburgh, Pennsylvania , VA New England Mental Illness, Research, Education and Clinical Center (MIRECC), VA Pennsylvania Healthcare System, Pittsburgh; Geisel School of Medicine, Department of Psychiatry, Dartmouth College; Concord, New Hampshire, Collaborative Design for Recovery and Health, Nashua, New Hampshire, Northeast Program Evaluation Center, Department of Veterans Affairs, West Haven, Connecticut, National Center for PTSD Evaluation Division, Department of Veterans Affairs, West Haven, Connecticut; Riverside Community Mental Health, Dedham, Massachusetts; Rutgers University, Department of Biology, Camden, New Jersey; Families in Trauma and Recovery, PeerLed, Lived Experience Social Enterprise, Fife Renewables Innovation Centre, Ajax Way, LEVEN, Fife, Scotland; Temple University, Department of Social and Behavioral Sciences, College of Public Health, Philadelphia, Pennsylvania; Geisel School of Medicine, Department of Psychiatry, Dartmouth College; Concord, New Hampshire, Collaborative Design for Recovery and Health, Nashua, New Hampshire; Geisel School of Medicine, Department of Psychiatry, Dartmouth College; Concord, New Hampshire , Collaborative Design for Recovery and Health, Nashua, New Hampshire; The Bridge, New York, New York; Department of Psychiatry, Massachusetts General Hospital, Boston, Massachusetts, Harvard Medical School, Boston, Massachusetts; VA New England Mental Illness, Research, Education and Clinical Center (MIRECC), VA Connecticut Healthcare System, West Haven, Connecticut, Department of Psychiatry, School of Medicine, Yale University, New Haven, Connecticut; Johns Hopkins University School of Medicine, Baltimore, Maryland; VA New England Mental Illness, Research, Education and Clinical Center (MIRECC), VA Pennsylvania Healthcare System, Pittsburgh; Collaborative Design for Recovery and Health, Nashua, New Hampshire; Pat Deegan and Associates, LLC; Uncompagre Band of the Ute Indian Tribe from the Uintah and Ouray Agency in Fort Duchesne, Utah; Seven Counties Services, Inc, Louisville, Kentucky; Forbes Senior Contributor and, Rebel Talent; The National Council for Mental Wellbeing, Washington, DC; The National Council for Mental Wellbeing, Washington, DC; The National Council for Mental Wellbeing, Washington, DC; Pittsburgh Mercy, University of Pittsburgh School of Medicine, Pittsburgh, Pennsylvania; The Danya Institute, Silver Spring, Maryland; Office of Mental Health and Suicide Prevention, Department of Veterans Affairs, Washington, DC, Duke University School of Medicine, Durham, North Carolina; L.E.A.R.N. (Lived Experience Academic and Research Network) Queensland, Australia; National Alliance on Mental Illness, Arlington, Virginia; Geisel School of Medicine, Department of Psychiatry, Dartmouth College; Concord, New Hampshire, Collaborative Design for Recovery and Health, Nashua, New Hampshire, The Dartmouth Institute for Health Policy and Clinical Practice, Geisel School of Medicine, Dartmouth College, Lebanon, New Hampshire; Community Care Behavioral Health, UPMC Insurance Services Division, Pittsburgh, Pennsylvania; OPROMAMER, Rwanda, Africa; Collaborative Design for Recovery and Health, Nashua, New Hampshire, Massachusetts Department of Mental Health, Boston, Massachusetts; Health Science, University of Stavanger, Stavanger, Norway; Collaborative Design for Recovery and Health, Nashua, New Hampshire, Massachusetts Department of Mental Health, Boston, Massachusetts

## Abstract

**IMPORTANCE:**

People with serious mental illness (SMI), defined as a diagnosis of schizophrenia spectrum disorder, bipolar disorder, or disabling major depressive disorder) die approximately 10 to 25 years earlier than the general population.

**OBJECTIVE:**

To develop the first-ever lived experience–led research agenda to address early mortality in people with SMI.

**EVIDENCE REVIEW:**

A virtual 2-day roundtable comprising 40 individuals convened on May 24 and May 26, 2022, and used a virtual Delphi method to arrive at expert group consensus. Participants responded to 6 rounds of virtual Delphi discussion via email that prioritized research topics and agreement on recommendations. The roundtable was composed of individuals with lived experience of mental health and/or substance misuse, peer support specialists, recovery coaches, parents and caregivers of people with SMI, researchers and clinician-scientists with and without lived experience, policy makers, and patient-led organizations. Twenty-two of 28 (78.6%) of the authors who provided data represented people with lived experiences. Roundtable members were selected by reviewing the peer-reviewed and gray literature on early mortality and SMI, direct email, and snowball sampling.

**FINDINGS:**

The following recommendations are presented in order of priority as identified by the roundtable participants: (1) improve the empirical understanding of the direct and indirect social and biological contributions of trauma on morbidity and early mortality; (2) advance the role of family, extended families, and informal supporters; (3) recognize the importance of co-occurring disorders and early mortality; (4) redefine clinical education to reduce stigma and support clinicians through technological advancements to improve diagnostic accuracy; (5) examine outcomes meaningful to people with an SMI diagnosis, such as loneliness and sense of belonging, and stigma and their complex relationship with early mortality; (6) advance the science of pharmaceuticals, drug discovery, and choice in medication use; (7) use precision medicine to inform treatment; and (8) redefine the terms *system literacy* and *health literacy.*

**CONCLUSIONS AND RELEVANCE:**

The recommendations of this roundtable are a starting point for changing practice and highlighting lived experience–led research priorities as an option to move the field forward.

## Introduction

People with serious mental illness (SMI), such as schizophrenia spectrum disorder, bipolar disorder, or major depressive disorder, die 10 to 25 years earlier than the general population, irrespective of geography, race and ethnicity, health care systems, or financing.^1–3^ Poor diet, smoking, and physical inactivity are the prominent contributors to preventable early mortality in this population in the US.^4^ Over the past 3 decades, health promotion and self-management interventions and national initiatives (eg, Substance Abuse and Mental Health Services Administration 10 × 10 campaign)^5^ have promoted health behavior change to address modifiable risk factors in people with SMI.^6^ Additionally, research agendas to address this health inequity, including a group gathered by the World Health Organization (WHO) and another, titled the Blueprint to Address Early Mortality, have been developed.^7^ Despite these efforts, the mortality gap between those with SMI and the general population is increasing.^2,3,8^ It is critical to consider alternative approaches to prevention and treatment.

Community engagement with people with SMI can inform research to make it relevant to target populations, potentially producing greater uptake and better clinical outcomes.^9^ A new paradigm led by individuals with lived experience may reveal innovative avenues to address this health disparity. This report presents a lived experience–led research agenda to address early death in people with SMI.

## Methods

We participated in and are members of the Early Mortality in People with SMI Roundtable, which convened virtually on May 24 and 26, 2022. The roundtable is a collaborative committed to addressing the early mortality health disparity in people with SMI through patient-centered research (Table). Twenty-two of 28 of the participants (78.6%) represented people with lived experiences.

Roundtable members were selected by reviewing the peer-reviewed and gray literature on early mortality and SMI. Two of us (K.L.F. and R.W.) conducted a Google Scholar search using variations of search terms: *early mortality* and *SMI* and *death, premature death, schizophrenia, bipolar* and then emailed authors identified through this search. Those identified members recommended additional members using a snowball sampling framework.^10^ Patient partners were identified through direct email to partners of the Collaborative Design for Recovery and Health, an international group of patients and scientists. The collaborative uses community-based participatory research with people with SMI to coproduce solutions to address community-identified needs. For example, this group developed the PeerTECH app designed to support the delivery of evidence-based practices by peer support specialists.^11^

Session 1 began with a welcome and introductions, followed by a discussion on the scientific understanding of early mortality documented in the peer-reviewed literature. Next, one of us (D.J.) presented a story with a gap^12^ to elicit gaps in the research. The story with a gap technique includes 2 contrasting pictures of before and after situations. Another one of us (R.W.) implemented “go wild” prompts (ie, “wouldn’t it be good if…?”) and reverse brainstorming to generate ideas about the causes of early mortality. Immediately following the first day, 3 of us (K.L.F., S.L., and P.G.) drafted a research agenda based on the gaps identified during the first day of the virtual convening. All authors were emailed a shared document of the draft research agenda 24 hours before the next session and were encouraged to review the materials. Session 2 began with a review of the draft research agenda in a shared document. One of us (R.W.) used multivoting, ranking, and problem-solving methods to help members refine language and ideas.

After the convening, all members received a web link to a shared document. Next, the roundtable used a virtual Delphi method—an empirically supported process used to arrive at expert group consensus^13^—to reach consensus (Figure). Participants responded to 6 rounds of virtual Delphi via email that prioritized research topics and agreement on recommendations. Participants were asked to rank items via the anonymous survey to allow for nuance in opinion and avoid the pitfalls of making binary choices. After each round, all responses were aggregated by one of us (K.L.F.) and shared with the group via email, and an anonymous survey link was sent until a 100% consensus of the authors was achieved.

## Results

The following recommendations are presented in order of priority as identified by virtual convening members.

### Improve the Empirical Understanding of the Direct and Indirect Social and Biological Contributions of Trauma on Morbidity and Early Mortality

#### Background

Compared with the general population, people with SMI experience a substantially higher incidence and prevalence rates of trauma—between 51% and 98% of people in the public mental health system have experienced trauma at some point.^14,15^ Trauma is defined as a psychological or emotional response to a disturbing or distressing experience^14^ (eg, singular traumas, such as interpersonal violence, intergenerational trauma, and iatrogenic trauma, and ongoing traumas, such as food insecurity, racism, and discrimination). According to a longitudinal study of 4462 male veterans, the experience of trauma, in particular posttraumatic stress disorder, contributes to poor physical, emotional, and mental health and early mortality.^16–21^ For example, people may develop harmful coping mechanisms, such as unhealthy eating, substance abuse, or self-harm. Biologically, the experience of trauma can also create inflammation in the body, which can lead to the development and exacerbation of conditions such as pulmonary and metabolic diseases.^22^

Current recommendations, including those put forth by a group gathered by the WHO,^23^ did not discuss trauma as a factor in early mortality. The Blueprint to Address Early Mortality^24^ discussed the role of child abuse as a social determinant of health, which is an important type of traumatic event that may impact individuals throughout their lifespan.

#### Recommendations

We make the following recommendations. Existing or new interventions designed to increase the lifespan of people with SMI (ie, self-management and health promotion) currently do not address the role of trauma in early mortality.^6^ Furthermore, research agendas, including those put forth by the WHO^23^ and *Lancet*,^24^ only recognized child abuse as a social determinant of health. Interventions need to recognize the role of trauma and its impact on the lifespan and potential impact on health behaviors. A strategic approach may be using community-engaged research to adapt interventions with widespread uptake, such as peer support,^20^ to include intervention components that focus on addressing trauma (eg, emotional CPR). Methodologically rigorous studies can then integrate complexity science to use multimodal treatment to explore the role of trauma and its effect on early mortality and potential impact on health behaviors. Complexity science is physical, biological, and social systems research to understand complex systems.^25^

### Advance the Role of Family, Extended Families, and Informal Supporters

#### Background

We adopted a culturally informed definition of family, which includes immediate family, extended families (eg, grandparents, aunts, and uncles), chosen families (ie, people who have intentionally chosen to support each other regardless of blood or marriage), and informal supporters (eg, neighbors, school, and church). Family members and informal supporters provide unpaid care.^26^

Current recommendations from the WHO recognize family interventions as an evidence-based practice to support individualized treatment or assist with interventions that focus on self-management or recovery.^23^ Delivering family psychoeducation to caregivers and patients with schizophrenia spectrum disorders has shown improvements in caregivers’ functioning and burden and patient outcomes.^27^ While important, to our knowledge, the impact of interventions that include family broadly defined on early mortality has not been explored scientifically. Future scientific exploration could examine the best practices to support engagement and collaboration using technology to promote the reach of these interventions.

#### Recommendations

We make the following recommendations. First, when possible, include family, extended family, and informal supporters in existing interventions to support their loved one in the community between intervention sessions. Such involvement may affect outcomes, such as early psychosis and relapse and rehospitalization rates, and it could improve social functioning and employment rates and increase hope and empowerment. For example, family psychoeducation (ie, an evidence-based approach designed to help families and informal supporters of people with SMI better understand mental health challenges) has been associated with great reductions in relapse and rehospitalization rates, higher employment rates, greater social functioning, and higher levels of hope and empowerment among people with an SMI diagnosis.^28^ Subpopulation analyses should explore race and ethnicity, age, and gender. Second, consider the bidirectional impact of caregiving on the care partner. Examining family interventions, such as family member respite, family consultation, system navigation, family member training, and family psychoeducation—and their role in optimizing outcomes associated with early mortality—can support the advancement of this unpaid workforce.

### Recognize the Importance of Co-occurring Disorders and Early Mortality

#### Background

Individuals with co-occurring disorders commonly have at least 1 mental disorder as well as a substance abuse disorder.^29^ In 2020, for adults aged 18 years or older, people with an SMI diagnosis had the following prevalence rates of substance use disorders: 39.2% for marijuana, 47.8% for illicit drugs, 11.6% for opioids, 30.9% for binge alcohol use, and 37.4% for tobacco or vaped nicotine.^29^ Individuals with co-occurring disorders are at an increased risk of premature drug death, suicide, and violent victimization.^29^ Despite the high comorbidity of substance use and mental health challenges in the general population,^30^ the mental health and substance abuse treatment systems remain mostly separate and create barriers to treatment for people with SMI. Many people with an SMI diagnosis have difficulty navigating both the mental health and substance abuse systems.^31^

#### Recommendations

We make the following recommendations. First, research on integrated treatment and comparison with sequential and parallel models of care is needed. The Blueprint to Address Early Mortality recommends the following: integrated interventions as the highest standard and a clear referral policy between mental health and substance use treatment services in sequential or parallel treatments.^24^ In contrast, a systematic review of the literature on a general (non-SMI) population^24^ has reported preliminary evidence of this treatment system; however, a superiority or an inferiority trial has never been conducted with a fully powered sample. Research in this area could compare an integrated co-occurring disorders system with sequential or parallel treatment programs.

Second, a research area to consider is smoking cessation postintervention withdrawal in settings other than studies. Although most smokers with SMI want to quit^32^ and cessation is most likely when both pharmacologic and psychosocial treatment are used,^33^ cessation rates are low, and most smokers with SMI relapse months after treatment,^33^ suggesting room for improvement in cessation treatments. An initial step may be the integration of qualitative research guided by implementation science to explore barriers and facilitators to cessation postintervention outside of study settings.

### Redefine Clinical Education to Reduce Stigma and Support Clinicians Through Technological Advancements to Improve Diagnostic Accuracy

#### Background

Historically, people with an SMI diagnosis have received inadequate physical health care.^4^ Despite having twice as many health care encounters as the general population, individuals with SMI receive fewer screenings, prescriptions, diagnoses, and surgical procedures.^34^ In response, health homes were developed to coordinate physical health care by integrating primary health care within community-based behavioral health care. Health homes have resulted in increased preventive screening; however, patients’ cardiometabolic outcomes associated with early mortality rarely improved.^20^ Current recommendations indicate a need for increased screening for medical conditions alongside integrated care initiatives, including appropriate, timely interventions, care coordination, and collaboration with social welfare involvement.^23^ Existing recommendations also specify the need to develop local, national, and global health policy around SMI; provide equitable access to universal health care; improve the use of medical investigation and treatment; and prevent diagnostic overshadowing (ie, physical conditions inaccurately characterized as being the result of a mental condition).^24^

#### Recommendations

We make the following recommendations. First, social workers, nurses, home health aides, psychologists, primary care practitioners, and psychiatrists need exposure to people with SMI in residency via coursework in formal education or through continuing education and internships. This exposure can include (1) instruction on physical health, mental health, and social health complexity; (2) therapeutic techniques to support the integration of the patient’s voice into clinical encounters (eg, active listening); and (3) education on the history of the mental health system in the US from asylums to nontrauma-informed care and deinstitutionalization and how this experience may affect clinical encounters. Second, technology can be incorporated into clinical care to support clinicians in universal screening and the accuracy of diagnosis and treatment. For example, audio recordings of medical appointments between people diagnosed with an SMI and clinical practitioners can incorporate natural-language processing to record conversations longitudinally. Audio data can then be coded for self-reported symptoms and verified with medical records to study diagnostic accuracy.

### Examine Outcomes Meaningful to People With an SMI Diagnosis, Such as Loneliness and Sense of Belonging, and Stigma and Their Complex Relationship With Early Mortality

#### Background

The prevailing understanding of the cause of early mortality in people with SMI has been defined as poor health behaviors (eg, diet, smoking, and exercise); however, limited knowledge exists on the association between poor health behaviors and early mortality in people with SMI. McGinnis and Foege^35^ reported the causes of death in the US were predominately due to poor health behaviors. This study was conducted with the general population and excluded people with SMI, yet these findings have been generalized to people with SMI. Furthermore, health behaviors in comparison with social determinants of health or patient-identified risk factors for early mortality may have a direct or mechanistic effect on early mortality and/or engagement in health behaviors.

Current recommendations from the WHO and the Blueprint to Address Early Mortality cite traditional social determinants of health, such as poverty, poor education, unemployment, homelessness, and childhood abuse as having an impact on mortality.^23,24^ The WHO recommends stigma-reduction programs for people with SMI. The Blueprint to Address Early Mortality recommends that stigma within health care and among practitioners be addressed and that neuromotor adverse drug reactions, which carry stigma, be dealt with by research into psychotropic interventions.^24^

#### Recommendations

We make the following recommendations. First, we recommend the development of a lived experience–powered research network^36^ (ie, coproduced database that is governed by people with SMI and supports data collection directly from persons with an SMI). This research network can support examining outcomes meaningful to people with SMI in combination with biological, psychological (health behaviors), and social (loneliness) variables throughout the lifespan. This database can determine a longevity phenotype to guide intervention and drug development and an SMI exposome to allow for the examination of the relationships between the person, their environment, and health disparities. The database infrastructure can be designed to conduct population health research to accelerate the pace of science.

### Advance the Science of Pharmaceuticals, Drug Discovery, and Choice in Medication Use

#### Background

Appropriate administration of antipsychotic medications through continuous medication treatment and proper dosing reduces excess mortality in persons with SMI.^37,38^ For example, the Clinical Antipsychotic Trials of Intervention Effectiveness Study, a nationwide public health–focused clinical trial, outlines a prescription protocol for people with schizophrenia at greater baseline risk for cardiometabolic events.^39^ However, despite this advancement, the leading cause of death among people with an SMI diagnosis is cardiovascular disease, which is associated with a higher relative risk of dyslipidemia, smoking, diabetes, and obesity.^40^

#### Recommendations

We make the following recommendations. First, decision aids or decision support could be integrated with decision-making to support informed patient decision-making regarding medication use.^41^ The Antipsychotic Medication Decision Aid^41–43^ is an example of a decision-aid intervention used during psychiatric visits. Development and implementation of additional decision-support tools for other drugs with adverse effects (eg, lithium) may expand our empirical understanding of the role of these aids in early mortality. Second, successful drug discovery may require exploring stem cell research, novel protein-drug conjugate modeling and assays, and advancement toward precision medicine.^44–46^ One compelling proof of concept that links pertinent physiologic models of human disease to drug development endeavors is to advance the testing of experimental therapeutics using patient-specific induced pluripotent stem cell models.^47^ This strategy, an advancement toward precision medicine,^47^ has the potential to close the loop of discovery that is propelled by human illness biologic factors at every step of the process. In the future, such innovations hold considerable potential as a contribution to developing clinically effective drugs that address the issue of early death in people with an SMI diagnosis.

### Use Precision Medicine to Inform Treatment

#### Background

We note the prevailing definition of SMI that groups people with heterogeneous diagnoses, which include clinical and biological variability,^24,48^ as a homogeneous population. While transdiagnostic approaches aim to produce scalable strategies,^7^ this type of categorization likely explains the observed heterogeneity of effect sizes for the same treatment between different people with SMI who have the same diagnosis.^48,49^ Analytic approaches based on big data, combined with recent scientific discoveries on the dynamic relationships between biopsychosocial, medical, and environmental determinants of SMI, afford the opportunity of personally tailored programs through precision medicine. Broadly defined, precision medicine is treatment that is tailored to each patient. Novel tools based on an individual’s biopsychosocial signature can enhance treatment decisions to produce the best possible outcome.

Scientific studies commonly group people with an SMI diagnosis as a homogeneous group; rather, the overlap in polygenic risk between bipolar disorder and schizophrenia for some patients, bipolar and major depressive disorder for others, and bipolar disorder and attention-deficit hyperactivity disorder for others may have implications for mental health treatment outcomes.^24^ As such, empirically exploring outcomes by SMI type in fully powered samples may elucidate nuances to individual differences and best modifiable treatment practices for improved outcomes.^24^ Researchers call for transdiagnostic approaches to better account for individual-level differences (eg, gender, cultural, and racial and ethnic identity) to lead to tailored and scalable strategies. To date, it is not known whether individually tailored approaches in comparison with transdiagnostic approaches lead to better outcomes and/or scalable strategies.

#### Recommendations

We make the following recommendations. First, people with SMI represent a heterogeneous group and the exploration of the Sequential, Multiple Assignment Randomized Trial (SMART)^50^ research method is warranted. The SMART design is a useful technique for building stepped-care models and just-in-time adaptive interventions.^50^ SMART trials allow for rerandomization based on an individual’s response. The SMART research method can allow for the field to progress more rapidly and perhaps reduce the time for effective interventions to be implemented. Second, use all data sources to inform personalized treatment options. Because of the nature of SMI, patients often have frequent encounters with the health care system that produce vast amounts of data. Accurate use of these data through predictive analytics is paramount to identifying beneficial treatment options before the trial of any interventions.

### Redefine the Terms *System Literacy* and *Health Literacy*

#### Background

Health literacy is the degree to which an individual has the capacity to find, understand, and use health-related information to inform health-related decisions and behaviors.^51^ Being health literate also includes being able to place the health of one’s family and community into context, understanding the factors that influence health, and being equipped to address them. Up to 66% of people with SMI have suboptimal health literacy, compared with 26% of those in the general population.^51^ Low health literacy among people with an SMI diagnosis can affect engagement in interventions and increase the risk for hospitalization and other adverse health outcomes.^51,52^ The potential low level of health literacy in people with SMI, as well as their increased risk of preventable chronic diseases associated with early mortality, call for advancing the health literacy of this group.^52^ The WHO recommends focusing interventions on lower socioeconomic groups with lower health literacy.^23^

#### Recommendations

We make the following recommendations. Universal design should be incorporated into paper-based or technology-based interventions, peer-delivered or clinician-delivered interventions, and dissemination strategies. Universal design is “the design of products and environments to be usable by all people, to the greatest extent possible, without the need for adaptation or specialized design.”^53^ Universal design is incorporated to support the readability and learnability of health-related information. It is accessible to people with varying literacy levels and impairments in vision and hearing, including people with SMI.^54^ This framework can improve usability and learnability (eg, by reducing memory and cognitive loads).

## Discussion

This lived experience–led research is a starting point to support interested parties in advancing the science of early mortality in people with SMI. The identified recommendations are a departure from what is known, and may offer a potentially viable path to extending the life span of people with SMI.

### Limitations

While we tried to account for various early mortality causes, not all causes of mortality were considered (eg, suicidality) as the roundtable was a 2-day event with select people with varying perspectives. However, this convening is an important step in including people with a lived experience in the conversation.

## Conclusions

The recommendations presented herein offer a starting point for changing practice and highlighting lived experience–led research priorities as an option to move the field forward. If we recognize the value of disrupting our current research and make changes in how we design and conduct research, select interventionists, and structure our health care systems, medical education systems, and dissemination, we may impact the early mortality health disparity for persons with SMI.

## Figures and Tables

**Figure.**
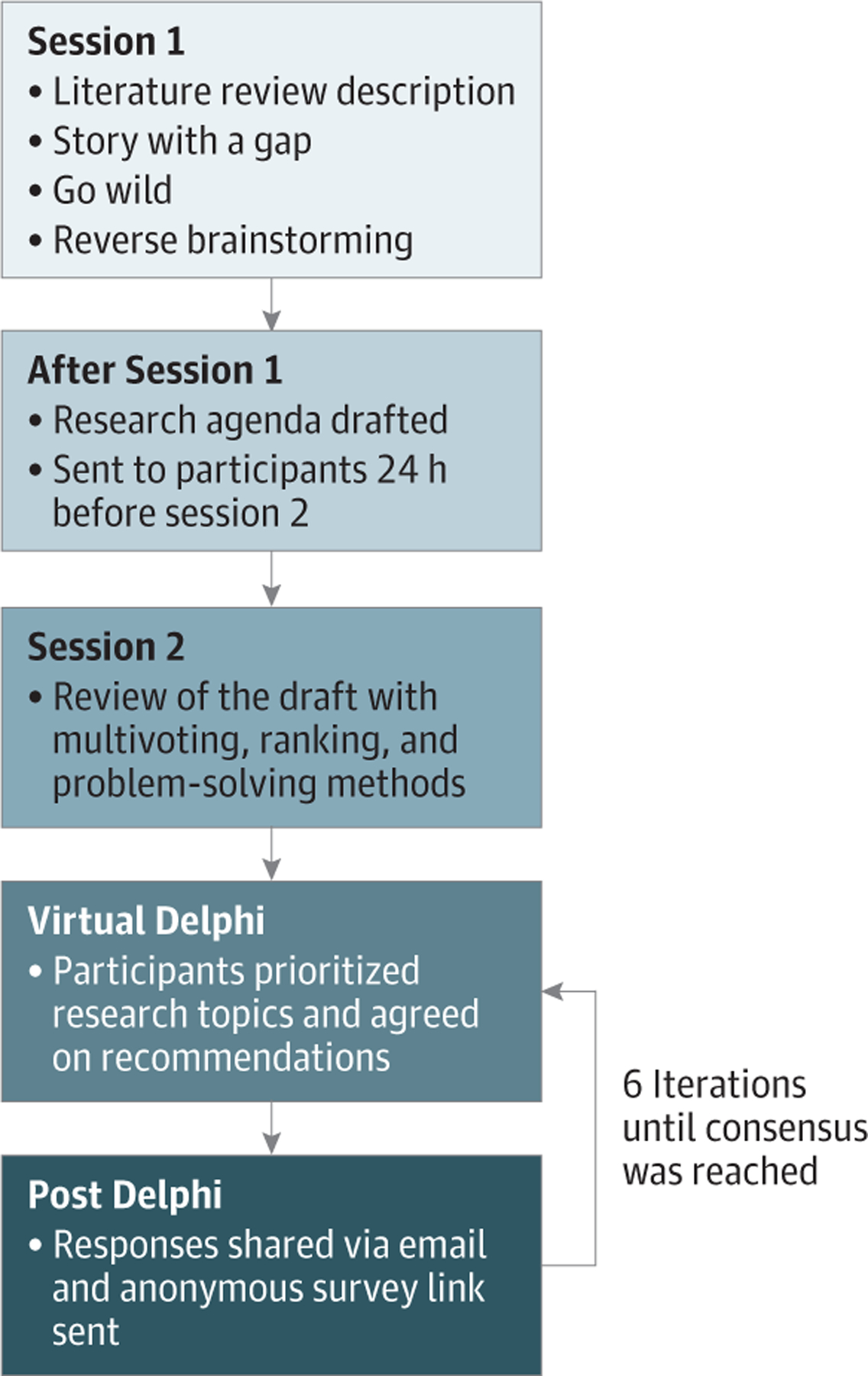
Schematic Overview of Consensus and Virtual Delphi Processf

**Table.** Demographic Information of Roundtable Members

Sociodemographic characteristic^a^	No. (%) (N = 28)
Gender	
Male	8 (28.6)
Female	20 (71.4)
Race	
African American	4 (14.3)
American Indian or Alaska Native	1 (3.6)
Asian	1 (3.6)
Hispanic/Latino	2 (7.1)
White	20 (71.4)
Background (nonmutually exclusive)	
Person with a lived experience	22 (78.6)
Caregiver of a person with SMI	7 (25.0)
Policy maker	3 (10.7)
Certified peer support specialists	6 (21.4)
Payer system	1 (3.6)
Clinician	10 (35.7)
Basic scientist	2 (7.1)
Implementation scientist	6 (21.4)
Intervention scientist	9 (32.1)
Justice involved	1 (3.6)
Advocate	1 (3.6)
Executive leader	2 (7.1)

Abbreviation: SMI, serious mental illness.

a Data not reported from 12 roundtable members.

## References

[1] Hayes JF, Miles J, Walters K, King M, Osborn DPJ. A systematic review and meta-analysis of premature mortality in bipolar affective disorder. Acta Psychiatr Scand 2015;131(6):417–425. doi:10.1111/acps.12408 25735195 PMC4939858

[2] Saha S, Chant D, McGrath J. A systematic review of mortality in schizophrenia: is the differential mortality gap worsening over time? Arch Gen Psychiatry. 2007;64(10):1123–1131. doi:10.1001/archpsyc.64.10.1123 17909124

[3] Walker ER, McGee RE, Druss BG. Mortality in mental disorders and global disease burden implications: a systematic review and meta-analysis. JAMA Psychiatry. 2015;72(4):334–341. doi:10.1001/jamapsychiatry.2014.2502 25671328 PMC4461039

[4] DE Hert M, Correll CU, Bobes J, Physical illness in patients with severe mental disorders. I. Prevalence, impact of medications and disparities in health care. World Psychiatry. 2011;10(1):52–77. doi:10.1002/j.2051-5545.2011.tb00014.x 21379357 PMC3048500

[5] Kobrin M Promoting wellness for better behavioral and physical health. SAMSHA. Accessed April 25, 2023. https://mfpcc.samhsa.gov/ENewsArticles/Article12b_2017.aspx

[6] Whiteman KL, Naslund JA, DiNapoli EA, Bruce ML, Bartels SJ. Systematic review of integrated general medical and psychiatric self-management interventions for adults with serious mental illness. Psychiatr Serv 2016;67(11): 1213–1225. doi:10.1176/appi.ps.201500521 27301767 PMC5089924

[7] Firth J, Siddiqi N, Koyanagi A, The Lancet Psychiatry Commission: a blueprint for protecting physical health in people with mental illness. Lancet Psychiatry. 2019;6(8):675–712. doi:10.1016/S2215-0366(19)30132-4 31324560

[8] Lawrence D, Kisely S, Pais J. The epidemiology of excess mortality in people with mental illness. Can J Psychiatry. 2010;55(12):752–760. doi:10.1177/070674371005501202 21172095

[9] Haldane V, Chuah FLH, Srivastava A, Community participation in health services development, implementation, and evaluation: A systematic review of empowerment, health, community, and process outcomes. PLoS One. 2019;14(5):e0216112. doi:10.1371/journal.pone.0216112 31075120 PMC6510456

[10] Parker C, Scott S, Geddes A. Snowball sampling. In: Atkinson P, Delamont S, Cernat A, Sakshaug JW, Williams RA, eds. SAGE Research Methods Foundations. SAGE Publications Ltd; 2020.

[11] Fortuna K, Barr P, Goldstein C, Application of community-engaged research to inform the development and implementation of a peer-delivered mobile health intervention for adults with serious mental illness. J Particip Med 2019;11(1):e12380. doi:10.2196/12380 32095314 PMC7039401

[12] Narayan D, Rietbergen-McCracken J. Participation and Social Assessment: Tools and Techniques. World Bank Publications; 1998.

[13] Dalkey N, Helmer O. An experimental application of the DELPHI method to the use of experts. Manage Sci 1963;9(3):458–467. doi:10.1287/mnsc.9.3.458

[14] Mueser KT, Goodman LB, Trumbetta SL, Trauma and posttraumatic stress disorder in severe mental illness. J Consult Clin Psychol 1998;66(3):493–499. doi:10.1037/0022-006X.66.3.493 9642887

[15] Switzer GE, Dew MA, Thompson K, Goycoolea JM, Derricott T, Mullins SD. Posttraumatic stress disorder and service utilization among urban mental health center clients. J Trauma Stress. 1999;12(1):25–39. doi:10.1023/A:1024738114428 10027140

[16] Substance Abuse and Mental Health Services Administration. SAMHSA’s concept of trauma and guidance for a trauma-informed approach. July 2014. Accessed April 26, 2023. https://ncsacw.acf.hhs.gov/userfiles/files/SAMHSA_Trauma.pdf

[17] Substance Abuse and Mental Health Services Administration. Trauma-informed care in behavioral health services. 2014. Accessed October 28, 2022. https://www.ncbi.nlm.nih.gov/books/NBK207192/24901203

[18] Trémeau F, Antonius D, Malaspina D, Goff DC, Javitt DC. Loneliness in schizophrenia and its possible correlates.: an exploratory study. Psychiatry Res 2016;246:211–217. doi:10.1016/j.psychres.2016.09.043 27721059

[19] Ong AD, Rothstein JD, Uchino BN. Loneliness accentuates age differences in cardiovascular responses to social evaluative threat. Psychol Aging. 2012;27(1):190–198. doi:10.1037/a0025570 22004517

[20] Fortuna KL, DiMilia PR, Lohman MC, Systematic review of the impact of behavioral health homes on cardiometabolic risk factors for adults with serious mental illness. Psychiatr Serv 2020;71(1):57–74. doi:10.1176/appi.ps.201800563 31500547 PMC6939136

[21] Boscarino JA. Psychobiologic predictors of disease mortality after psychological trauma: implications for research and clinical surveillance. J Nerv Ment Dis 2008;196(2):100–107. doi:10.1097/NMD.0b013e318162a9f5 18277217

[22] Michopoulos V, Powers A, Gillespie CF, Ressler KJ, Jovanovic T. Inflammation in fear- and anxiety-based disorders: PTSD, GAD, and beyond. Neuropsychopharmacology. 2017;42(1):254–270. doi:10.1038/npp.2016.146 27510423 PMC5143487

[23] Zhu T Challenges of psychiatry drug development and the role of human pharmacology models in early development—a drug developer’s perspective. Front Psychiatry. 2021;11:562660. doi:10.3389/fpsyt.2020.562660 33584358 PMC7873432

[24] Coombes BJ, Markota M, Mann JJ, Dissecting clinical heterogeneity of bipolar disorder using multiple polygenic risk scores. Transl Psychiatry. 2020;10(1):314. doi:10.1038/s41398-020-00996-y 32948743 PMC7501305

[25] Mitleton-Kelly E, Paraskevas A, Day C. Handbook of Research Methods in Complexity Science: Theory and Applications. Edward Elgar Publishing Limited; 2018. doi:10.4337/9781785364426

[26] Miles P, Brown N, Grealish M, Berg JVD, Pina V, Franz J. The Wraparound Milwaukee Fieldbook. Vol 1. Supporting Effective Child and Family Team; 1996.

[27] Mueser KT, Achtyes ED, Gogate J, Mancevski B, Kim E, Starr HL. Telehealth-based psychoeducation for caregivers: the family intervention in recent-onset schizophrenia treatment study. JMIR Ment Health. 2022;9(4): e32492. doi:10.2196/32492 35436231 PMC9055490

[28] Cohen AN, Glynn SM, Murray-Swank AB, The family forum: directions for the implementation of family psychoeducation for severe mental illness. Psychiatr Serv. 2008;59(1):40–48. doi:10.1176/ps.2008.59.1.40 18182538

[29] SAMHSA. Substance use disorder treatment for people with co-occurring disorders: treatment improvement protocol tip 42. 2020. Accessed April 25, 2023. https://store.samhsa.gov/sites/default/files/SAMHSA_Digital_ Download/PEP20-02-01-004_Final_508.pdf

[30] Mueser KT, Noordsy DL, Fox L, Wolfe R. Disulfiram treatment for alcoholism in severe mental illness. Am J Addict 2003;12(3):242–252. doi:10.1111/j.1521-0391.2003.tb00652.x 12851020

[31] Jones CM, McCance-Katz EF. Co-occurring substance use and mental disorders among adults with opioid use disorder. Drug Alcohol Depend. 2019;197:78–82. doi:10.1016/j.drugalcdep.2018.12.030 30784952

[32] Tulloch HE, Pipe AL, Clyde MJ, Reid RD, Els C. The quit experience and concerns of smokers with psychiatric illness. Am J Prev Med 2016;50(6):709–718. doi:10.1016/j.amepre.2015.11.006 26711162

[33] Evins AE, Cather C, Laffer A. Treatment of tobacco use disorders in smokers with serious mental illness: toward clinical best practices. Harv Rev Psychiatry. 2015;23(2):90–98. doi:10.1097/HRP.0000000000000063 25747922 PMC4460830

[34] Weinstein LC, Stefancic A, Cunningham AT, Hurley KE, Cabassa LJ, Wender RC. Cancer screening, prevention, and treatment in people with mental illness. CA Cancer J Clin 2016;66(2):134–151. doi:10.3322/caac.21334 26663383 PMC4783271

[35] McGinnis JM, Foege WH. Actual causes of death in the United States. JAMA. 1993;270(18):2207–2212. doi:10.1001/jama.1993.03510180077038 8411605

[36] PCORnet patient-powered research network phase ll: ImproveCareNow Network. July 15, 2015. Accessed February 27, 2023. https://www.pcori.org/research-results/2015/pcornet-patient-powered-research-network-phase-ll-improvecarenow-network

[37] Rubino A, Sanon M, Ganz ML, Association of the US Food and Drug Administration antipsychotic drug boxed warning with medication use and health outcomes in elderly patients with dementia. JAMA Netw Open. 2020;3(4):e203630. doi:10.1001/jamanetworkopen.2020.3630 32343351 PMC7189225

[38] Liu NH, Daumit GL, Dua T, Excess mortality in persons with severe mental disorders: a multilevel intervention framework and priorities for clinical practice, policy and research agendas. World Psychiatry. 2017;16 (1):30–40. doi:10.1002/wps.20384 28127922 PMC5269481

[39] Swartz MS, Stroup TS, McEvoy JP, What CATIE found: results from the schizophrenia trial. Psychiatr Serv 2008;59(5):500–506. doi:10.1176/ps.2008.59.5.500 18451005 PMC5033643

[40] Rossom RC, Crain AL, O’Connor PJ, Effect of clinical decision support on cardiovascular risk among adults with bipolar disorder, schizoaffective disorder, or schizophrenia: a cluster randomized clinical trial. JAMA Netw Open. 2022;5(3):e220202. doi:10.1001/jamanetworkopen.2022.0202 35254433 PMC8902652

[41] Zisman-Ilani Y, Shern D, Deegan P, Continue, adjust, or stop antipsychotic medication: developing and user testing an encounter decision aid for people with first-episode and long-term psychosis. BMC Psychiatry. 2018;18(1):142. doi:10.1186/s12888-018-1707-x 29788933 PMC5963160

[42] Zisman-Ilani Y, Gorbenko KO, Shern D, Elwyn G. Comparing digital vs paper decision aids about the use of antipsychotic medication: client, clinician, caregiver and administrator perspectives. Int J Person Centered Med July 13, 2017. Accessed October 28, 2022. http://www.ijpcm.org/index.php/IJPCM/article/view/618

[43] Zisman-Ilani Y, Hurford I, Bowen A, Salzer M, Thomas EC. Evaluating the feasibility of a decision aid to promote shared decision making among young adults with first-episode psychosis: protocol for a pilot study. Pilot Feasibility Stud 2021;7(1):22. doi:10.1186/s40814-020-00757-0 33431018 PMC7798319

[44] Meng XY, Zhang HX, Mezei M, Cui M. Molecular docking: a powerful approach for structure-based drug discovery. Curr Comput Aided Drug Des 2011;7(2):146–157. doi:10.2174/157340911795677602 21534921 PMC3151162

[45] Watson JL, Juergens D, Bennett NR, Broadly applicable and accurate protein design by integrating structure prediction networks and diffusion generative models. bioRxiv. Preprint published online December 14, 2022. doi:10.1101/2022.12.09.519842

[46] Haggarty SJ, Silva MC, Cross A, Brandon NJ, Perlis RH. Advancing drug discovery for neuropsychiatric disorders using patient-specific stem cell models. Mol Cell Neurosci 2016;73:104–115. doi:10.1016/j.mcn.2016.01.011 26826498 PMC5292010

[47] Haggarty SJ, Karmacharya R, Perlis RH. Advances toward precision medicine for bipolar disorder: mechanisms & molecules. Mol Psychiatry. 2021;26(1):168–185. doi:10.1038/s41380-020-0831-4 32636474 PMC10290523

[48] Dacquino C, De Rossi P, Spalletta G. Schizophrenia and bipolar disorder: the road from similarities and clinical heterogeneity to neurobiological types. Clin Chim Acta 2015;449:49–59. doi:10.1016/j.cca.2015.02.029 25704299

[49] Liang SG, Greenwood TA. The impact of clinical heterogeneity in schizophrenia on genomic analyses. Schizophr Res 2015;161(2–3):490–495. doi:10.1016/j.schres.2014.11.019 25496659 PMC4308487

[50] Collins LM, Murphy SA, Nair VN, Strecher VJ. A strategy for optimizing and evaluating behavioral interventions. Ann Behav Med 2005;30(1):65–73. doi:10.1207/s15324796abm3001_8 16097907

[51] Degan TJ, Kelly PJ, Robinson LD, Deane FP, Smith AM. Health literacy of people living with mental illness or substance use disorders: a systematic review. Early Interv Psychiatry. 2021;15(6):1454–1469. doi:10.1111/eip.13090 33254279

[52] Krishan S, von Esenwein SA, Druss BG. The health literacy of adults with severe mental illness. Psychiatr Serv 2012;63(4):397-397. doi:10.1176/appi.ps.20120p397 22476312

[53] Connell BR, Jones ML, Mace RL, The Principles of Universal Design. Version 2.0; 1997.

[54] Fortuna KL, Kadakia A, Cosco TD, Guidelines to establish an equitable mobile health ecosystem. Psychiatr Serv 2023;74(4):393–400. doi:10.1176/appi.ps.202200011 36377370 PMC11398716

